# Predicting Absorption-Distribution Properties of Neuroprotective Phosphine-Borane Compounds Using In Silico Modeling and Machine Learning

**DOI:** 10.3390/molecules26092505

**Published:** 2021-04-25

**Authors:** Raheem Remtulla, Sanjoy Kumar Das, Leonard A. Levin

**Affiliations:** 1 Department of Ophthalmology and Visual Sciences, McGill University, Montreal, QC H4H 3S5, Canada; raheem.remtulla@mail.mcgill.ca; 2 Drug Discovery Core, Research Institute, McGill University Health Centre, Montreal, QC H4A 3J1, Canada; sanjoykumar.das@muhc.mcgill.ca; 3 Department of Neurology and Neurosurgery, McGill University, Montreal, QC H3A 2B4, Canada

**Keywords:** neuroprotection, pharmacokinetics, neural networks, phosphine-borane compounds

## Abstract

Phosphine-borane complexes are novel chemical entities with preclinical efficacy in neuronal and ophthalmic disease models. In vitro and in vivo studies showed that the metabolites of these compounds are capable of cleaving disulfide bonds implicated in the downstream effects of axonal injury. A difficulty in using standard in silico methods for studying these drugs is that most computational tools are not designed for borane-containing compounds. Using in silico and machine learning methodologies, the absorption-distribution properties of these unique compounds were assessed. Features examined with in silico methods included cellular permeability, octanol-water partition coefficient, blood-brain barrier permeability, oral absorption and serum protein binding. The resultant neural networks demonstrated an appropriate level of accuracy and were comparable to existing in silico methodologies. Specifically, they were able to reliably predict pharmacokinetic features of known boron-containing compounds. These methods predicted that phosphine-borane compounds and their metabolites meet the necessary pharmacokinetic features for orally active drug candidates. This study showed that the combination of standard in silico predictive and machine learning models with neural networks is effective in predicting pharmacokinetic features of novel boron-containing compounds as neuroprotective drugs.

## 1. Introduction

Neurodegenerative diseases affecting the optic nerve are a common cause of visual loss [1,2]. The loss of vision can be rapid, e.g., from inflammatory, ischemic, or traumatic causes, or chronic, from compressive, glaucomatous, toxic, nutritional, or hereditary causes [2]. Typical clinical presentations include loss of visual acuity, visual field defects, and dyschromatopsia [2]. In all cases the primary mechanism is damage to retinal ganglion cell (RGC) axons contained within the optic nerve [3]. Axonal damage induces RGC somatic death via a variety of mechanisms, including signaling via reactive oxygen species (ROS), particularly superoxide, based on studies increased levels in neurons after injury and that ROS scavengers prevent RGC death [4,5]. One mechanism for ROS signaling is that highly oxidative environments increase the formation of disulfide bonds between cystine side chains and cause cellular damage [6,7,8]. Support for this mechanism is based on the neuroprotective properties of disulfide reducing agents both in vitro and in vivo [6,7].

Phosphine-borane complexes are redox-active drugs which have been studied as potential clinical neuroprotectants [4,5,8,9]. These molecules activate the extracellular signal-regulated kinase-1/2 cell survival pathway and preserve reduced cystine side chains [1,8,10,11]. Studies using triphenyl phosphine and aryl disulfides have suggested that phosphorus atoms in phosphine-borane molecules act as a nucleophile in an S_N_2 nucleophilic substitution reaction [10]. As well, in assays using an intracellular dithiol reporter indicate that phosphine-borane compounds are able to cleave intracellular disulfide bonds and unpublished data from our lab indicates these compounds cleave interchain disulfide bonds [10].

**Figure 1:**
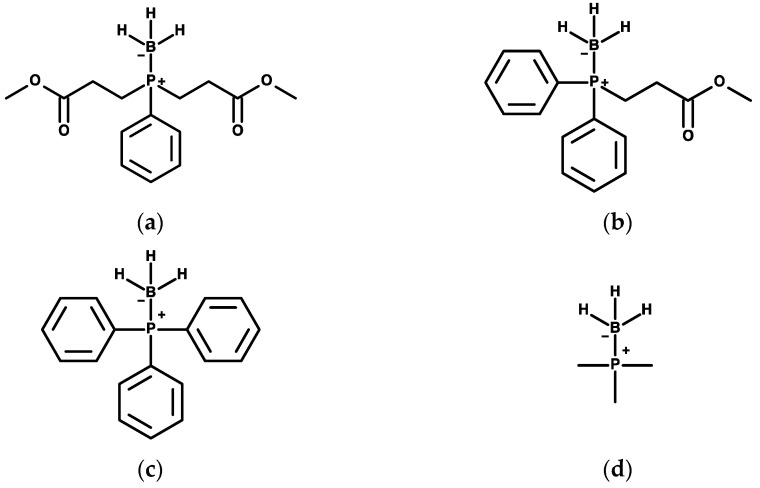
Phosphine-borane structures. Chemical structures of (**a**) PB1, (**b**) PB2 (**c**) PB-Ph, and (**d**) PB-Me.

Phosphine-borane compounds contain a borane group which protects the reactive phosphorus atom of the compound from reducing chemical bonds indiscriminately [8,10]. Thus, the boronated compounds acts as the pro-drug for the phosphorus-exposed compounds. Once deboronated, the compounds cleave disulfide bonds via an S_N_2 nucleophilic substitution reaction and then are oxidized. After oxidation, these compounds are unable to cleave disulfide bonds. There are three steps in phosphine-borane metabolism (1) pro-drug (phosphine-borane complex); (2) active drug (phosphine); and (3) drug metabolites (phosphine-oxides). Four known phosphine-borane compounds known to our lab are demonstrated in Figure 1.

Understanding the pharmacokinetic features of these different steps is essential to determine their clinical value and will help in the development of future phosphine-borane compounds. Unfortunately, there is limited pharmacological data available on phosphine-borane compounds. Advancements in computational methods have allowed for the rapid assessment of these features. Unfortunately, these techniques often rely on large databases of compound structures that typically lack boron atoms and thus cannot be used to test phosphine-borane complexes. Without these methodologies, computational methodologies cannot be used to evaluate pertinent pharmacokinetic features of phosphine-borane processes, which would hamper the drug discovery process. However, physiochemical characteristics can be predicted by employing computational methodologies based on machine learning and help in determining their potential use in clinical settings. Machine learning methodologies allow for the development of automated mode building by identifying patterns in existing data. The advantage of this is that it allows for reliable models to be trained with relative ease. Using existing in silico methodologies physiochemical characteristics of phosphine-borane compounds and their metabolites were predicted and absorption-distribution characteristics of non-boron phosphine-borane metabolites. Using these in silico properties neural networks were trained to predict the absorption-distribution characteristics phosphine-borane compounds.

## 2. Results

### 2.1. Absorption

#### 2.1.1. Cellular Permeability

**Figure 2:**
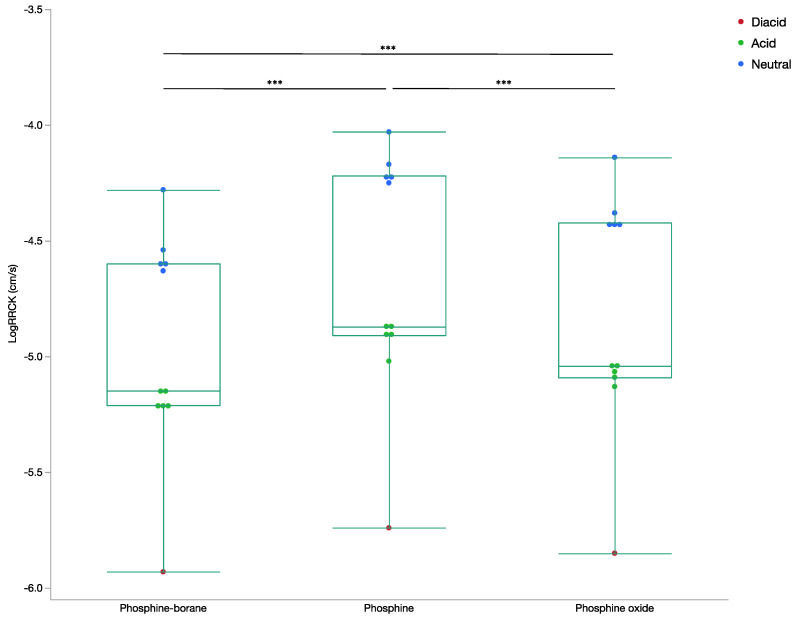
Box plot of LogRRCK permeability by compound step LogRRCK (cm/s) was determined from Maestro for all phosphine-borane, phosphine, and phosphine oxide compounds. Ion states for all compounds are included. (ANOVA, *** *p* < 0.001).

The mean logRRCK for phosphine-boranes compounds was −4.96 ± 0.47 cm/s (110 nm/s). For phosphine compounds it was −4.65 ± 0.52 cm/s (224 nm/s), and for phosphine oxides it was −4.82 ± 0.50 cm/s (151 nm/s). These values were all statistically significantly different from each other (*p* < 0.0001; Figure 2). Although the compounds had moderate membrane permeability, phosphine compounds were ~100 nm/s faster than phosphine-borane compounds (*p* < 0.0001). As expected, diacids and acids had lower permeability than their neutral counterparts. All non-hydrolyzed compounds had a logRRCK value greater than the suggested permeability cut-off of −5 cm/s, indicating good cellular permeability [12,13]. None of the hydrolyzed phosphine-borane and phosphine oxide compounds had logRRCK greater than −5 cm/s, indicating that ester hydrolysis of these bonds may prevent membrane permeability of these compounds.

#### 2.1.2. Oral Absorption

The neural network was able to successfully predict human oral absorption after 1000 epochs of training, with a 0.979 concordance with actual results in the training set and 0.895 in the test set. The network performance is demonstrated in Table 1 and the data division between the test and training sets is demonstrated in Table 2. The absolute error was minimal in each set, with a mean absolute error rate of 2.3% in the training set and 5.7% in the test set, an indication of minimal overfitting. The average correlation between the oral absorption over 20 additional iterations of training was 0.989 in the training set and 0.844 in the test set. There was no statistically detectable difference between the selected neural network predicted results and the Maestro QikProp predicted values (*p* = 0.656). Furthermore, the correlation between the presented network and QikProp predictions was high for both phosphine and phosphine oxide compounds (0.937 and 0.992 respectively). The results for the network performances are presented in Table 2. To verify the validity of the presented network, the network was used to predict the oral absorption of boric acid. Boric acid has an oral absorption between 81–95%, the presented network predicted the oral absorption of boric acid to be 81.1% [14,15].

**Figure 3:**
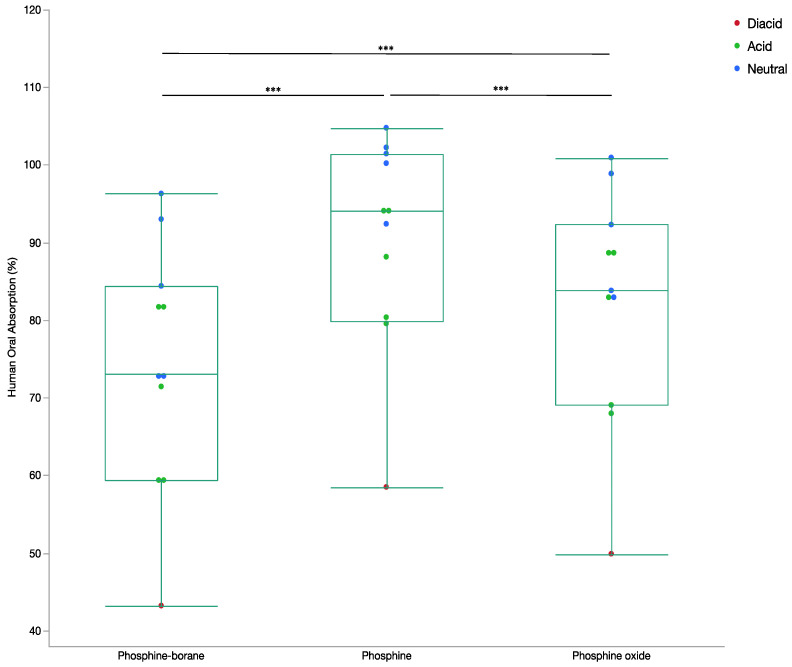
Box and whisker plot of percentage human oral absorption by compound step. The percentage human absorption determined from the presented neural network for all phosphine-borane, phosphine, and phosphine oxide compounds. Ion states for all compounds are included. (ANOVA, *** *p* < 0.001).

The mean permeability for the phosphine-borane compounds was 74.2 ± 17.7% indicating a moderate permeability. Permeability for the phosphine compounds was 90.5 ± 13.6% and 82.4 ± 15.0% for phosphine oxide compounds, both indicating high permeability. All permeabilities compounds were statistically different from one another (*p* < 0.0001; Figure 3). According to the Maestro knowledge base greater than 80% is considered high absorption and lower than 20% is considered low absorption [16]. These findings suggest that phosphine-borane compounds are predicted to passively cross the gut-blood barrier [13].

#### 2.1.3. Blood-Brain Barrier Absorption

The neural network learned to successfully predict the logBB in 1000 epochs with a 0.994 correlation with actual results in the training set and 0.968 in the test set. The absolute error was minimal in each set, with a mean absolute error rate of 0.14 in the training set and 0.08, in the test set, suggesting that overfitting was unlikely. Repeated network training identified an average correlation in the training set of 0.995 and 0.940 in the test set. There was no statistical difference between the QikProp predicted logBB and the neural network predicted logBB, with a probability value of 0.606. Among phosphine compounds the correlation between the QikProp and network predicted logBB was 0.994 and among phosphine oxide compounds the correlation was 0.995. The network predicted the logBB for boric acid to be 0.36 (actual 0.31) and for bortezomib to be −2.46 (actual −1.36 to −1.15) [17,18].

**Figure 4:**
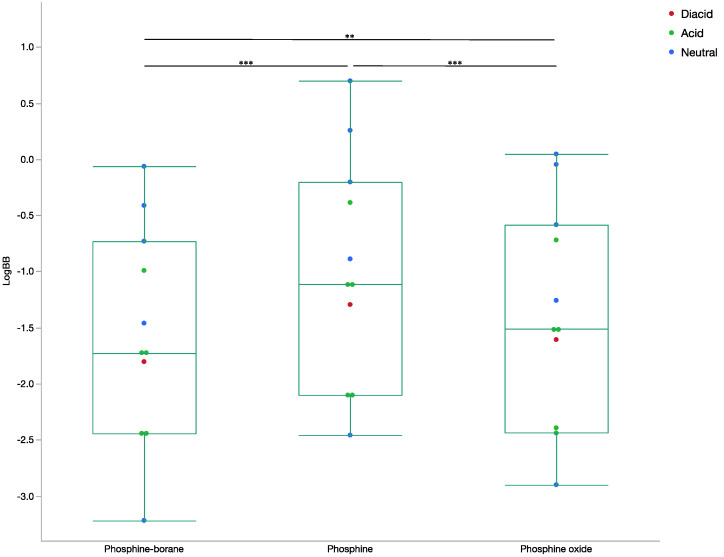
Box and whisker plot of LogBB by compound step. The LogBB determined from the presented neural network for all phosphine-borane, phosphine, and phosphine oxide compounds. Ion states for all compounds are included. (ANOVA, *** *p* < 0.001, ** *p* < 0.05).

The mean logBB for phosphine-borane compounds was −1.55 ± 0.95, for phosphines it was −0.97 ± 1.00, and for phosphine oxides it was −1.36 ± 0.98 (*p* < 0.0001; Figure 4). Negative logBB values indicate non-favourable penetration and positive values demonstrate high penetration. The Maestro knowledge base reports most drugs have logBB values ranging from −3.0 to 1.2, however this includes both CNS permeable and non-permeable drugs [16]. The predicted logBB values were negative, indicating a lower CNS concentration compared to the concentration within the rest of the body compartment. The negative logBB values indicate that a high serum concentration of phosphine-borane compounds would be needed to ensure a therapeutic concentration is reached within the central nervous system.

### 2.2. Distribution

#### 2.2.1. Octanol/Water Coefficient

The octanol/water distribution coefficients, with a correlation between the predicted values of MarvinSketch and QikProp of 0.948, were determined. There was a significant difference (*p* < 0.0001) between the octanol/water coefficient of phosphine–borane complexes, phosphines, and phosphine oxides. For phosphine boranes the mean MarvinSketch logP was 2.16 ± 3.10, for phosphines the mean was 3.82 ± 2.87, and for phosphine oxides the mean was 3.04 ± 3.10. All values were consistent with lipophilic compounds with logP values less than the Lipinski rule cut-off of 5 and within the range of logP values suggested by the Maestro knowledge base [16].

#### 2.2.2. LogK_HSA_

The maximum momentum constant was reached, and training was completed for the logK_HSA_ assay prediction network at 463 epochs. The correlation for logK_HSA_ in the training set was 0.916 and 0.958 in the test set. There was little evidence of overfitting, with the mean absolute error rate being minimal in each set (0.37 in the training set and 0.32 in the test set). Repeated network training resulted in the mean correlation of predicted to actual logKHSA values in the training set of 0.937 and 0.819 in the test set. There was no statistically detectable difference between the selected neural network and the Maestro QikProp predicted logK_HSA_ (*p* = 0.947). Furthermore, the correlation between the presented network and QikProp predictions was high for both phosphine and (r = 0.989) phosphine oxide compounds (r = 0.977). The network predicted logK_HSA_ values of bortezomib, crisaborole and varborbactam were −1.46 (experimental protein binding 0.69), 0.03 (experimental protein binding 1.51) and −0.76 (experimental protein binding −0.31) [19,20,21].

**Figure 5:**
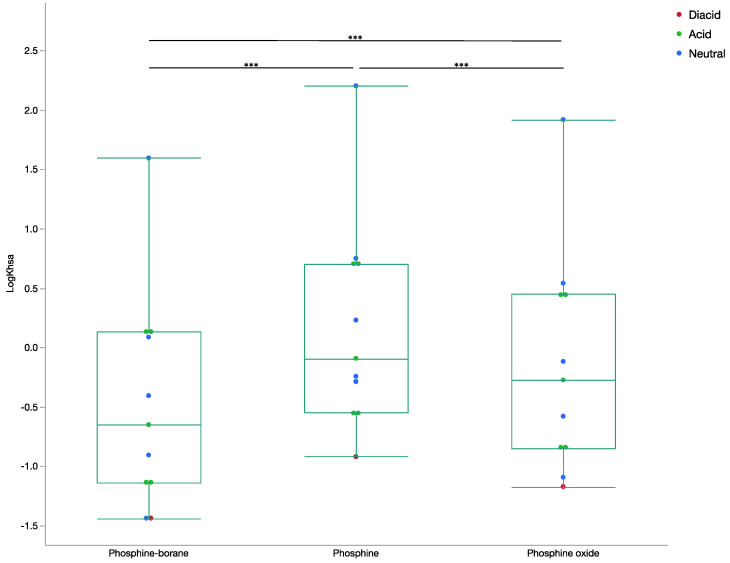
Box and whisker plot of LogK_HSA_ by compound step. The LogK_HSA_ determined from the presented neural network for all phosphine-borane, phosphine, and phosphine oxide compounds. Ion states for all compounds are included (ANOVA, *** *p* < 0.001).

The results indicated unfavorable albumin binding among phosphine-borane (−0.47 ± 0.91) and phosphine oxide (−0.14 ± 0.92) compounds and moderate binding among phosphine (0.18 ± 0.88) compounds (*p* < 0.0001; Figure 5). Compounds with negative logK_HSA_ have unfavorable albumin binding with most compounds ranging between −1.5 and 1.5 [16]. These findings indicated that phosphine-borane compounds are unlikely to be distributed through serum protein binding, however deboronated phosphine compounds may bind to albumin.

## 3. Discussion

We used machine learning to extend standard pharmacological tools previously limited to non-boron containing compounds to novel phosphine-borane compounds. To our knowledge, this is the first report where multiple pharmacokinetic features have been predicted for a novel class of compounds using a machine learning approach. The in silico findings indicate that phosphine-borane compounds are generally able to meet the necessary pharmacokinetic characteristics of pharmacologically active drugs. The compounds were able to moderately traverse passively the cell membrane, where the deboronated non-oxidized phosphines traverse the fastest. This indicates that the active drug (capable of cleaving disulfide bonds) can enter cells. Borane groups are removed when interacting with amine groups [22]. These amine groups are common in protein amino acids and could remove borane groups from phosphine-borane compounds. It is therefore possible to target specific cells through altering the interactions of the borane groups with amino acid side chains, such that the deboronated, and more membrane permeable phosphine, is present at the targeted cell. The moderate permeability of the phosphine oxide compounds indicates that cellular toxicity after bond cleavage is unlikely. Despite the zwitterion state of the initial prodrugs, most met the features necessary of an orally active medication outlined in Lipinski rules [23]. This is confirmed by the neural network prediction that the mean human oral absorption was 74.9%. Predictably, the less polar phosphine and phosphine oxides had a significantly higher absorption.

The predicted results from the blood-brain ratio neural network indicated that the phosphine compounds have an unfavorable concentration in the CNS. Thus, a high blood concentration of drug would be needed in order to effectively concentrate in the CNS. The compounds on average have an octanol/water partitioning coefficient consistent with drugs with high blood concentrations. This suggests that phosphine-borane compounds have features of compounds appropriate for systemic (e.g., oral or intravenous) administration. A different approach to ensuring high central nervous system concentration could also be to include small hydrophobic side chains instead of ester bonds. This would prevent hydrolysis to carboxylic acid groups that would make unfavorable blood brain barrier permeability. The highest logBB was among deboronated phosphine compounds, which would suggest CNS specificity could be increased by initiating deboronation near the blood-brain barrier.

Fortunately, there is existing data regarding blood brain barrier permeability of phosphine-borane compounds. Previous experimental data on PAMA-BBB artificial membranes made from porcine brain lipids demonstrated that PB2 was able to passively traverse the blood-brain barrier [11]. However, the PAMA-BBB results for PB1 contradicted the prediction from the neural network. It is possible that PB1 deboronated passively or by interacting with amine groups in the PAMA BBB model [11]. This would increase the permeability of the given species as deboronation of PB1 resulted in the deboronated phosphine compounds P1, which as predicted by the network had a higher logBB consistent with a higher blood brain barrier permeability. Phosphine-borane compounds have been shown to be deboronated by amine groups [11]. The predominate component (33.1% by weight) in artificial porcine membranes is phosphatidylethanolamine, which contains a primary amine group [24]. It is possible that the borane group from PB1 interacted with this amine, causing deboronation to the significantly more permeable deboronated PB1 and resulting in increased permeability [22,24,25]. It is also important to note that LogBB data is more suggestive of brain-blood barrier permeability because porcine cell membranes differ significantly from human cell membranes [24,25]. Since these values are negative and the network accuracy was high it can be assumed that compounds with these values do not undergo nonactive transport through the blood-brain barrier. 

The neural networks presented performed well except for the network used to model clearance. Given that all models which attempted to predict phosphine-borane outputs from QikProp were trained on deboronated or oxidized compounds, it is likely that their results were accurate for the phosphine-borane compounds studied. This is supported by the relations between the various predicted values, e.g., that the permeability was lowest for phosphine-borane compounds and highest for deboronated phosphine compounds across all metrics of permeability, including Maestro’s RRCK permeability, the network-predicted human absorption and logBB. The results from the logK_HSA_ network were consistent with the logP values determined by MarvinSketch and QikProp. It is likely that these neural networks had a high degree of accuracy because the logRRCK permeability was used as an input. Given that the networks were trained only on phosphine and phosphine oxide compounds, these networks may have limited use on compounds outside this class.

The input factors for neural network training came from multiple validated in silico models and the resultant networks attempted to predict features of the results from existing models. These existing models may have an underlying error and since we do not use experimental values in the inputs for the neural networks the model error may carry forward. Theoretically this should be negligible because the networks were validated against existing experimental results and the previously validated in silico methodologies. It is possible that the networks were able to account for existing error in the known in silico methodologies or that the existing error in these methodologies was negligible. Unfortunately, as with most machine learning applications the black box nature of the presented networks means that determining how the network deals with errors from underlying models is be challenging.

All networks that were studied demonstrated a high level of accuracy with limited overfitting when trained on phosphine and phosphine oxide compounds. The networks were also able to accurately predict the oral absorption of boric acid and the blood-brain barrier permeability of boric acid and bortezomib. These findings indicate that the network is able to predict pharmacokinetic features of boron-containing compounds. However, the predictions of albumin binding for bortezomib, crisaborole, and varborbactam were different from the experimentally determined serum protein binding values. It should be noted that the experimental values measured total protein binding and not human serum albumin binding. Albumin represents 62.5–70% of serum protein, and thus it is not surprising that the predicted logK_HSA_ were significantly lower than the experimentally predicted total protein binding [26]. The predicted logK_HSA_ values of crisaborole and varborbactam were approximately half of their experimentally predicted serum protein binding. In the context of the albumin-to-serum protein ratio, the predicted values are appropriate. However, there was a significant discrepancy between the experimental and predicted values for bortezomib. This may be explained by the significantly higher polar surface area of bortezomib. The polar surface area of bortezomib, as determined by MarvinSketch, was 164.9 angstroms, for the phosphine compounds it was 58.7 angstroms, and for the other known borane containing compounds the MarvinSketch predicted polar surface area was 84.2 angstroms. Thus, the logK_HSA_ network may only be effective for compounds with a lower polar surface area.

## 4. Materials and Methods

### 4.1. Compounds Tested

The pharmacokinetic properties of the following five phosphine–borane compounds and their metabolites were investigated: bis(3-propionic acid methyl ester) phenylphosphine-borane complex 1 (PB1), 3-propionic acid methyl ester) diphenylphosphine-borane complex (PB2), phenylphosphine-borane complex 19 (PB19), triphenylphosphine-borane complex (PB-Ph), and trimethylphosphine-borane complex (PB-Me). All known phosphine–borane compounds available to our lab and those existing in the literature were included for evaluation. No known phosphine–borane compounds were excluded from the analysis. Phosphine–borane complexes can undergo ester hydrolysis to form monoacids (PB1, PB2, or PB19) and diacids (PB1 or PB19). The chemical structures of tested phosphine–borane compounds are demonstrated in the supplemental material. Structures were directly drawn and optimized to their lowest energy states with Maestro (Schrodinger, New York, NY, USA; version 12.4.072) and MarvinSketch ChemAxon, Budapest, Hungary; version 17.10).

### 4.2. Absorption

Cellular permeability was determined with the Ralph Russ Canine Kidney (RRCK) cell-based assay Papp prediction available in Maestro 12.4.072. This physics-based model is used to predict RRCK (MDCK-LE) assay logPapp values [12,13,27]. Maestro’s rapid molecular properties tool QikProp was used to predict human oral absorption and the logarithmic brain/blood partition coefficient (logBB). QikProp compares compounds with a library of compounds to find those that are similar to them so that predictions on their physiochemical features can be determined. There are few compounds that include boron atoms in this database, and consequently boron-containing atoms cannot be evaluated by this model. For this reason, only the phosphine and phosphine oxide compounds were tested with QikProp. To predict the human oral absorption and logBB phosphine–borane compounds, neural networks were trained on the logRRCK and the permeability predicted with Maestro. MarvinSketch predicted logP and aqueous solubility and the molecular weight of the phosphine and phosphine oxide compounds. These features can be determined for compounds containing boron, and therefore a network using these features as inputs could be used to predict permeability features for the phosphine–borane compounds. Caco-2 cell permeability was attempted to be predicted as a marker for gut–blood barrier permeability. In non-borane-containing compounds QikProp was used to predict the Caco-2 cell permeability. Neural networks trained on this data demonstrated significant overfitting and therefore were excluded from the manuscript. MDCK permeability and CNS activity values were also predicted by QikProp in non-borane-containing compounds and neural networks were trained to predict these values in phosphine–borane compounds. However, these networks consistently were overfitted in the test sets and therefore were excluded from the manuscript.

### 4.3. Distribution

Octanol/water coefficient was determined with the MarvinSketch logP plugin using a consensus model based on the models derived from Klopman and Viswanadhan with the PHYSPROP database [28,29]. The default electrolyte concentrations were used due to their similarity to physiologic blood conditions [30,31]. Given the significance in drug distribution and a key component of Lapinski’s rules of five, logP values were compared among the stages of phosphine-borane compounds. The pH-dependent aqueous solubility curve was determined with the MarvinJS plugin at a pH of 7. LogPo/w and aqueous solubility was estimated in QikProp for all phosphine and phosphine oxide compounds. The logK_HSA_ was determined in QikProp for all non-boron containing compounds to estimate albumin binding. These results, along with the logRRCK, MarvinSketch logP, MarvinSketch aqueous solubility and molecular weight, were used to train a network to predict the logK_HSA_ for phosphine-borane compounds. To evaluate pharmacokinetic features of the presented drugs, we attempted to train neural networks to predict steady-state volume of distribution, clearance rate, blood-plasma ratio, and half-life using data available from Berezhkovskiy 2013. These networks either failed to accurately predict values in the training set or demonstrated significant overfitting and were therefore excluded from the manuscript.

### 4.4. Neural Network Training

**Figure 6:**
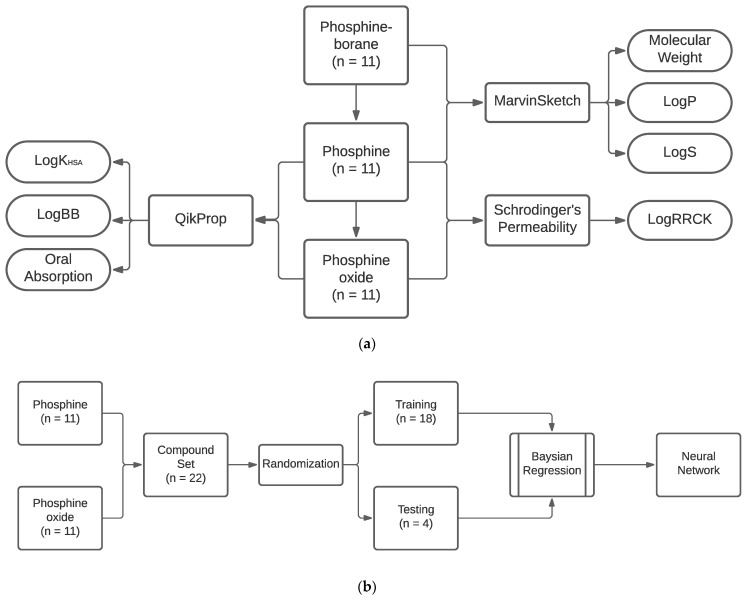
Process flow diagram for neural network training (**a**) Compound inputs to respective programs and predicted features. (**b**) Neural network training workflow.

The networks were constructed as two-layer feed-forward networks trained with a supervised learning approach using the Neural Network Toolbox available in MATLAB (MathWorks, Natick, MA, USA; version R2020a-9.8.0.1323502). The network was trained with a Bayesian regression with the FIT tool, which is well suited to developing generalizable networks on small datasets. Network training stopped at 1000 epochs or when the maximum momentum constant was reached. Using the standard randomization algorithm in MATLAB, compounds were randomly assigned to the testing of the training set. The internal validity of the trained neural networks was evaluated by determining the mean absolute error and by determining the correlation between the network output and the output of the standard methodology. We evaluated the error rates in the testing and training sets to ensure that no significant overfitting occurred. Furthermore, to ensure that an effective network was not generated stochastically, training was completed 20 additional times with distinct training and testing set divisions. The network with the lowest absolute error was presented. To ensure the external validity of the finalized presented network, the networks were used to predict features of known boron-containing compounds with existing experimentally determined pharmacological data and then compared. The process diagram for neural network training is demonstrated in Figure 6.

### 4.5. Statistics

Phosphine-borane compounds were compared to phosphine and phosphine oxide by one-way ANOVA with repeated measures within factors in JMP (SAS Institute, Cary, NC, USA; version 14.1.0) with all-pairs Tukey HSD comparisons for post hoc testing.

## 5. Conclusions

The in silico approach provided essential information on the pharmacokinetics of phosphine-borane compounds, demonstrating that despite the presence of a significant zwitterion, they have the features necessary for an orally active drugs. The used neural networks demonstrated that key pharmacokinetic features can be predicted with a machine learning approach Such networks could be implemented in future drug development of novel phosphine-borane compounds.

## Figures and Tables

**Table 1:** Neural network performance. Pearson correlation and absolute mean error between actual and predicted values for the training, test, and total set for all neural networks presented.

Measure	Phase	Oral Absorption	LogBB	LogK_HSA_
Correlation	Training	0.979	0.995	0.916
	Test	0.895	0.968	0.958
	Total	0.964	0.993	0.920
Mean Absolute	Training	2.33	0.14	0.37
Error	Test	5.73	0.08	0.32
	Total	2.95	0.13	0.36

**Table 2:** Comparison of training and test sets for all neural networks presented.

	Oral Absorption			LogBB				LogK_HSA_	
Measure	Training (SD)	Testing (SD)	Total	Training (SD)	Testing (SD)	Total	Training (SD)	Testing (SD)	Total
LogRRCK	−4.75 (0.53)	−4.70 (0.44)	0.87	−4.77 (0.52)	−4.62 (0.50)	0.61	−4.78 (0.51)	−4.55 (0.50)	0.42
Molecular Weight	324.4 (140.7)	273.3 (15.2)	0.49	334.7 (129.2)	226 (90.4)	0.13	327.0 (126.5)	261.3 (142.1)	0.37
LogP	3.52 (3.18)	3.02 (1.70)	0.77	3.83 (2.97)	1.59 (2.29)	0.17	3.45 (3.15)	3.34 (2.05)	0.95
LogS	−1.51 (3.88)	−1.20 (2.48)	0.88	−1.85 (3.83)	0.32 (1.94)	0.29	1.61 (3.80)	−0.73 (2.99)	0.67
Oral Absorption	86.0 (15.3)	90.5 (16.6)	0.61						
LogBB				−1.31 (1.02)	−0.47 (0.66)	0.13			
LogK_HSA_							−0.02 (1.07)	0.23 (0.82)	0.67

## Data Availability

All data including Maestro and MATLAB workspaces are available upon request.

## Sample Availability

Samples of the compounds are not available from the authors.
